# Association Analysis of Simple Sequence Repeat (SSR) Markers with Agronomic Traits in Tall Fescue (*Festuca arundinacea* Schreb.)

**DOI:** 10.1371/journal.pone.0133054

**Published:** 2015-07-17

**Authors:** Yanhong Lou, Longxing Hu, Liang Chen, Xiaoyan Sun, Yong Yang, Hongmei Liu, Qingguo Xu

**Affiliations:** 1 College of Agronomy, Hunan Agricultural University, Nongda Road, ChangSha City, Hunan, 410128, P.R. China; 2 Key Laboratory of Plant Germplasm Enhancement and Specialty Agriculture, Wuhan Botanical Garden, Chinese Academy of Sciences, Wuhan City, Hubei, 430074, P.R. China; 3 Golf College, Hunan International Economics University, Changsha, Hunan, 410205, P.R. China; National Institute of Plant Genome Research (NIPGR), INDIA

## Abstract

Tall fescue is widely used in temperate regions throughout the world as a dominant forage grass as well as a turfgrass, in pastoral and turf industry. However, the utilization of tall fescue was limited because of its leaf roughness, poor regeneration ability and poor stress resistance. New cultivars were desirable in modern pastoral industries exceed the potential of existing cultivars. Therefore, well understanding the agronomic traits and describing germplasms would help to overcome these constraints, and morphological evaluation of tall fescue germplasm is the key component in selecting rational parents for hybridization breeding. However, describing the morphological traits of tall fescue germplasm is costly and time-consuming. Fortunately, biotechnology approaches can supplement conventional breeding efforts for tall fescue improvement. Association mapping, as a powerful approach to identify association between agronomic traits and molecular markers has been widely used for enhancing the utilization, conservation and management of the tall fescue germplasms. Therefore, in the present research, 115 tall fescue accessions from different origins (25 accessions are cultivars; 31 accessions from America; 32 accessions from European; 7 accessions from Africa; 20 accessions from Asia), were evaluated for agronomic traits and genetic diversity with 90 simple sequence repeat (SSR) markers. The panel displayed significant variation in spike count per plant (SCP) and spike weight (SW). However, BCS performed the lowest CV among all the observed agronomic traits. Three subpopulations were identified within the collections but no obvious relative kinship (*K*) was found. The GLM model was used to describe the association between SSR and agronomic traits. Fifty-one SSR markers associated with agronomic traits were observed. Twelve single-associated markers were associated with PH; six single-associated markers were associated with BCS; eight single-associated markers were associated with SW; five single-associated markers were associated with SC; seven single-associated markers were associated with SCP; three single-associated markers were associated with SL. Especially, we observed that the genetic variation of SW was explained 11.6 % by M37 marker. It is interesting to observe that nine markers (M1, M2, M35, M54 marker was associated with both BCS and SC; M3, M4 markers were associated with BCS, SW, and SC; M19 marker was associated with both pH and PD, M40 marker was associated with both SCP and SW; and M193 marker was associated with both PH and SL) were associated with more than two agronomic traits. Notably, Branch count per spike (BCS) was explained by four markers (M1, M2, M3, and M4) exceeding 10 %. These identified marker alleles associated with agronomic traits could provide important information and markers for molecular-assisted breeding that facilitate the breeding process in tall fescue.

## Introduction

Tall fescue (*Festuca arundinacea* Scherb.) is an important hexaploid (2n = 6x = 42) perennial cool-season grass [1] with a genome size of 5.27 to 5.83 ×10^6^ kb [2]. Due to its excellent agronomic characteristics [3], tall fescue is the most widely planted grass in temperate regions throughout the world [4]. Natural populations are found in Europe, North-West Africa, North America, West and Central Asia [5]. Today, tall fescue is used extensively for forage in pastoral industry. However, leaf roughness, poor regeneration ability and poor stress resistance were the limiting factors for the widespread utilization of tall fescue [6, 7].

The requirement of elite cultivars in modern pastoral industries exceed the potential of existing cultivars in tall fescue, and well understanding the agronomic traits and describing germplasm would help to overcome this constraints [8]. Compared with other approaches, morphological evaluation is direct, inexpensive and easy. The knowledge of genetic variability for agronomic traits is the key component in selecting rational parents for hybridization breeding [9]. Plant height, flag leaf area, peduncle length, spike length, spikelet count per spike, spike count per plant and spike weight are the major components of plant yield selection criteria in breeding, which were observed to be significant genotypic variation in many crops [10, 11]. These variations might due to the effect of genotype and environment [12]. Previous researches indicated that significant genotypic variation was also observed in plant height, flag-leaf length, flag-leaf width among tall fescue accessions [13]. However, the negative situation that severe genetic erosion occurred in Tunisia reduced the substantial variation in tall fescue cultivars [14]. Therefore, in order to improve the tall fescue cultivars, it is important to detail the agronomic traits of tall fescue germplasm and then, efficiently utilize the genetic resources and broadening the gene pool [9].

Traditionally, describing the morphological traits of tall fescue germplasm is still costly and time-consuming, because a large sample size is essential prerequisite to provide a reasonable representation of overall genetic performance [15]. With the development of molecular markers, RFLPs [16], RAPD [17], AFLPs [18], and SSRs [19] was suitable for assessing the genetic diversity [20], and marker assisted selection [21]. SSRs markers have many advantages over other types of molecular markers, such as co-dominance, abundant in genomes, highly polymorphisms, locus specificity, good reproducibility and random distribution throughout the genome [21]. Recently, SSRs markers have been applied in traits and marker association of plants, such as kernel size and milling quality in wheat (*Triticum arstivum* L.) [22], flowering time of perennial ryegrass [23]. The availability of SSR markers in tall fescue was developed from an enriched genomic libraries [19], encouraged the utilization of SSR for cultivar identification and genetic diversity assessment in tall fescue.

Association mapping, based on linkage disequilibrium (LD), is a powerful technique to map molecular markers associated with phenotypic traits of interest based on natural populations, and offers an alternative method for QTL mapping [24, 25]. Association mapping utilizes diverse plant populations in detecting the correlations between certain alleles and specific traits more frequently than expected. As a promising approach for plant breeders, association mapping eliminates the main drawback of classical linkage analysis such as without prolonged, lingering and expensive generation of specific genetic populations, and unnecessary to development of new mapping populations. Furthermore, this approach can assess larger number of alleles and increase mapping resolution [26]. In recent years, association mapping have been successfully applied in rice [27], maize [28], barely [29], bean [30], sorghum [31], potato [32] and forage grass [23]. Knowledge on the location of the genetic determinants of the diversity may be useful for discovering new genes [33].

Considering the importance of association mapping for dissecting the complex quantitative agronomic traits, the objectives of the present study were to: (1) estimate genotype diversity among a core collection of 115 tall fescue accessions; and (2) analyze the association of SSR markers with various agronomic traits. The results of this study will help to utilize, conserve and manage the tall fescue germplasm effectively.

## Materials and Methods

### Plant materials and growth conditions

This research was carried out at Wuhan Botanical Garden, Chinese Academy of Science, Wuhan, China from 2012 to 2014. The origins of the 115 tall fescue accessions including 25 commercial cultivars were listed in Table A in S1 File.

Initially, a single seed from each accession was germinated on a filter paper which pre-soaked in distilled water in Petri dishes in May, 2012. Then, the Petri dishes were kept in the dark at 20°C until germination, and then placed in the growth container (LSC-339CF; Xingxing Group Co., Zhejiang, China) with 14 h photo-period, and light intensity of 300–500 μ mol photons m^-2^ s^-1^ natural sunlight. After two weeks of cultivation, all accessions were transferred to plastic containers (15 cm deep and 14 cm in diameter) which filled with a mixture of cultivation medium and sand (1: 1, v/v). Each accession was cloned multiple times by tillers to maintain genetic uniformity. All accessions were established in a walk-in growth room with daily maximum and minimum temperature of 24°C and 20°C, a 14-h photoperiod, and a light intensity of 300 μ mol photons m^−2^ s^−1^ at canopy height. All plants were watered daily to maintain soil volumetric water content at field capacity, and fertilized weekly with 300 mL of half-strength Hoagland’s solution [34]. Grasses were hand-clipped weekly at a 7 cm canopy height.

The field experiment plot was established in Sep, 2012, and initially treated with 49 kg N ha^-1^, 98 kg P ha^-1^, 98 kg K ha^-1^, and then covered sand with 2 cm-depth. All plants in plastic pots were transplanted to the experimental field in a 1.5×1.5 m-lattice with a randomized block plots with three replicates in October, 2012. The nutrition requirement of tall fescue was supplied by exogenous fertilizer applications. The compound fertilizer with N: P: K ratio 21: 6: 13 and urea were used alternatively with the amount of 49 kg N ha^-1^, and fertilized 7 times in 2013. All plants were mowed to the height of 10 cm after the data were collected each year, and no mowing was conducted at other time during the whole experimental period. In 2014, all accessions were fertilized with compound fertilizer in April, and were fertilized with urea in May to provide 49 kg N ha^-1^. Data were collected on June 4 to June 13 in 2013 and May 28 to Jun 7 in 2014. The experimental field was irrigated via perimeter pop-up gear-driven sprinkler heads positioned at 3.5 m, and irrigation plus rainfall to prevent the tall fescue accessions from wilt. When irrigated, water was applied to wet the entire root zone.

### Agronomic traits measurement

Agronomic traits were determined at the maturation stage in 2013 and 2014, including plant height (PH), spike length (SL), pulvinus distance (PD), spikelet count (SC), branch count per spike (BCS), spike count per plant (SCP), spike weight (SW). Data were collected from June 4 to June 13 in 2013 and from May 28 to Jun 7 in 2014.

Seven average plants from each accession per replication were selected to assess PH by averaged distance from the ground level to tip of these plants.

Four average plants from each accession per replicate were collected to determine the SL, PD, SC, BCS, SCP. SL was determined by averaged distance from the bottom to the tip of spike. PD was assessed by averaged distance from main spike neck to flag leaf pulvinus. SC, BCS and SCP were determined by averaged count of spikelet, branch and spike.

To determine the spike weight, four average plants from each accession per replication were also collected and detached from the plant. The spikes were weighed and then, averaged as SW of each accession.

### DNA isolation and SSR analysis

Young leaves (0.1 g) were selected and detached from the plant of each accession for total genomic DNA isolation using the cetyl trimethyl ammonium bromide (CTAB) method described by Xie et al. [35]. The quality of DNA was checked using 0.8% agarose gel electrophoresis, and the DNA concentration was measured using UV spectrophotometer [21]. In order to have a good coverage of the tall fescue genome, a set of 90 published genome-wide SSR markers [19, 36] mapped in 22 linkage groups in tall fescue were analyzed in all accessions (Table B in S1 File), and the forward primer sequence of markers were labeled with four fluorescent dyes of different colors [FAM (blue), HEX (green), TAMRA (yellow), and ROX (red)]. The polymerase chain reaction (PCR) described as follow: The final volume of 10 μl, containing 1×supplied Taq-buffer, 2.5 mM MgCl_2_, 200 μM dNTPs, 0.2 mM of each primer pair, 0.5 U of Taq DNA polymerase, and 30 ng of template DNA. DNA amplifications were performed in a 96-well My Cycler thermal cycler (Bio-Rad Inc., Hercules, CA, USA) using the following touchdown PCR protocol: 1 cycle of 10 min at 95°C; followed by 25 cycles of 50 s at 95°C, 50 s at 68°C with a decrease of 0.6°C in each consequent cycle, 60 s at 72°C. Another 15 cycles of 50 s at 95°C, 50 s at 54°C, 60 s at 72°C. The reaction ended with a 10 min extension at 72°C. ABI 3730 DNA Sequence (Applied Biosystems Inc., Foster City, CA, USA) was used in the PCR amplified fragments separation. Alleles were scored by GeneMarker 1.5 software (Soft Genetics, LLC, State College. PA, USA) and checked twice manually for accuracy. The bands detected for each microsatellite were recorded as a date matrix for the presence (1) and absence (0) of bands.

### Population Structure

The Bayesian model-based clustering method carried out in STRUCTURE 2.3.1 software [37] was employed to infer the population structure using 90 SSR markers and divided accessions into subpopulation. The length of burn-in period and the number of Markov Chain Monte Carlo (MCMC) replications after burn-in were all assigned at 100,000 with an admixture and allele frequencies correlated model. The structure was run ten times by setting pre-defined K (the number of population groups). The correct estimation of K was provided by joining the log probability of data [*LnP (D)*] from the STRUCTURE output and an ad hoc statistic ΔK, which was based on the second-order rate of change in *LnP(D)* between successive K values [38]. 15 independent runs were operated 100,000 interactions of each run after burn-in of 100,000 for a value of K setting from one to five.

### Associating mapping

Mean agronomic traits of the 7 quantitative traits (PH, SL, PD, SC, BCS, SCP, and SW) were subjected to association analysis with SSR loci, based on the whole set of 115 accessions used in this study.

Association analysis between the markers and the agronomic traits were performed based on the general linear model by using the software TASSEL 2.0.1 [39]. The data of polymorphism SSR locus was response variable, while the agronomic traits were independent variables. Markers were considered to be associated with the traits if the markers are significant (*P*<0.01).

### Statistical analysis

The experiment was arranged in a completely randomized block design with four replications. All agronomic traits data were averaged over two years. All data were performed with the SPSS statistical software package (version 20.0; SPSS, Chicago, IL, USA).

## Results

### Genetic variation in agronomic traits of tall fescue accessions

Significant genotypic variation in agronomic traits was determined for the tall fescue accessions (Table 1). Among all accessions, PH ranged from 34.3 cm to 176.1 cm, SL ranged from 13.0 cm to 44.2 cm, PD ranged from 24.5 to 88.8 cm, SC ranged from 36.8 to 183.6, BCS ranged from 9 to 18.8, SCP ranged from 6 to 137.2, and SW ranged from 3.87 to 76.5 (Table 1). SCP and SW exhibited greater coefficient of variation (CV) than other traits, with 55.68% and 48.28% respectively. In contrast, BCS performed lowest CV (13.93%) among all the observed traits. The CV of PH, SL, PD, SC, and SCP ranged from 19.92% to 28.23%. Performance and variation of agronomic traits of tall fescue was detailed in Lou et al. [40].

**Table 1:** Range of plant height (PH), spike length (SL), pulvinus distance (PD), spikelet count (SC), branch count per spike (BCS), spike count per plant (SCP), spike weight (SW) for 115 tall fescue accessions.

Traits	Maximum	Minimum	Mean	Std	CV%
**PH (cm)**	176.1	34.3	123.3	26.39	21.43
**SL (cm)**	44.2	13.0	29.8	5.93	19.92
**PD (cm)**	88.8	24.5	60.3	13.21	21.93
**SC**	183.6	36.8	89.2	25.19	28.23
**BCS**	18.8	9	12.6	1.76	13.93
**SCP**	137.2	6	49.6	27.63	55.68
**SW (g)**	76.5	3.87	28.9	13.97	48.28

Std. standard deviation

CV% coefficient of variation

### Genotype and Population structure analysis

**Fig 1:**
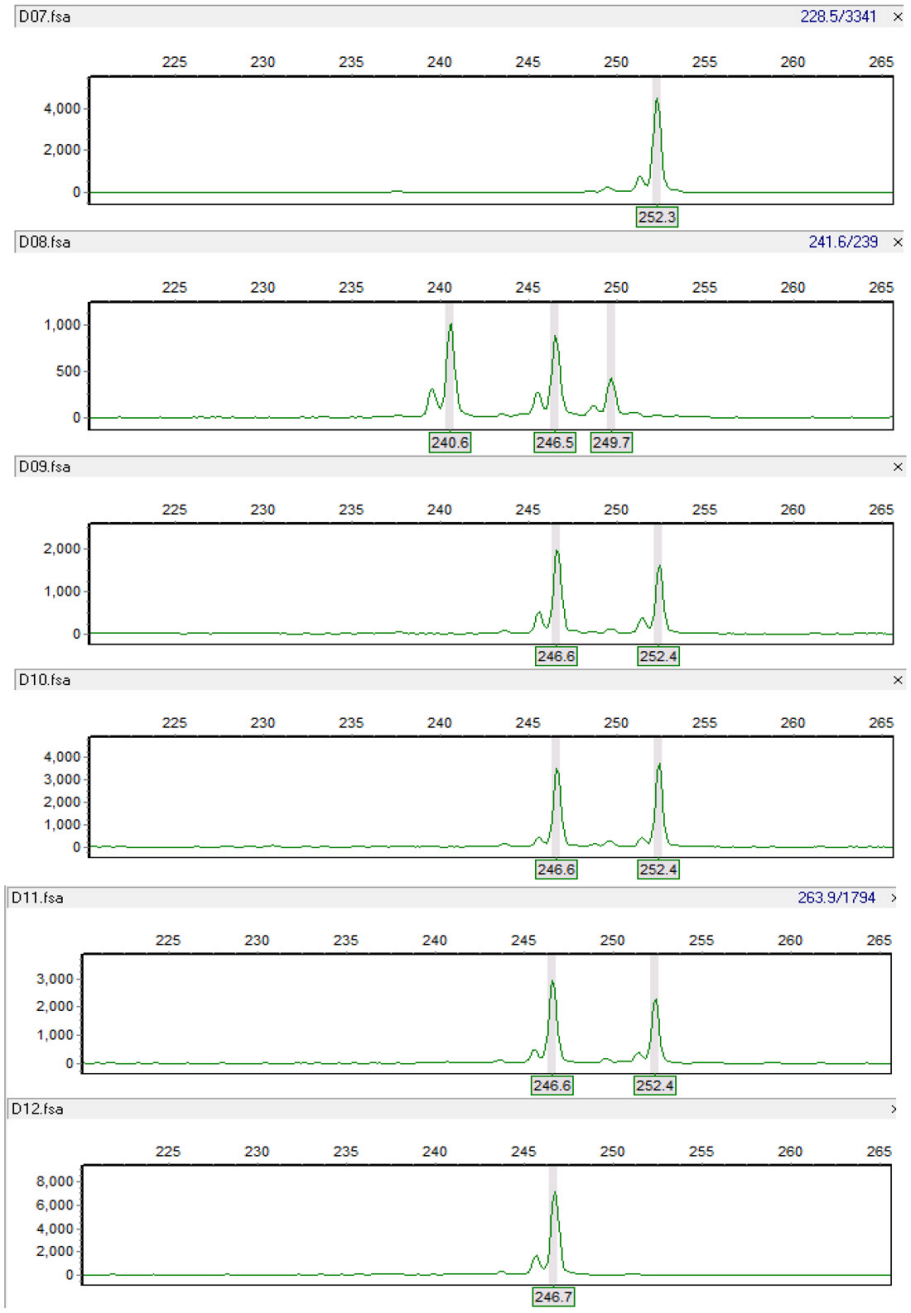
An example of amplification profiles of SSR marker M3.

A total of 1010 SSR alleles were obtained from the 90 SSR markers across the 115 tall fescue accessions with an average of 11.22 alleles per locus. The allele numbers of SSR marker varied from 3 to 27 alleles per marker, and all of the individuals were successfully distinguished by these bands (Fig 1). The genetic diversity of the 100 tall fescue accessions was at a relative lower level, in which average of *Nei*’s genetic diversity was 0.255, and average of polymorphism information content was 0.211 [41].

**Fig 2:**
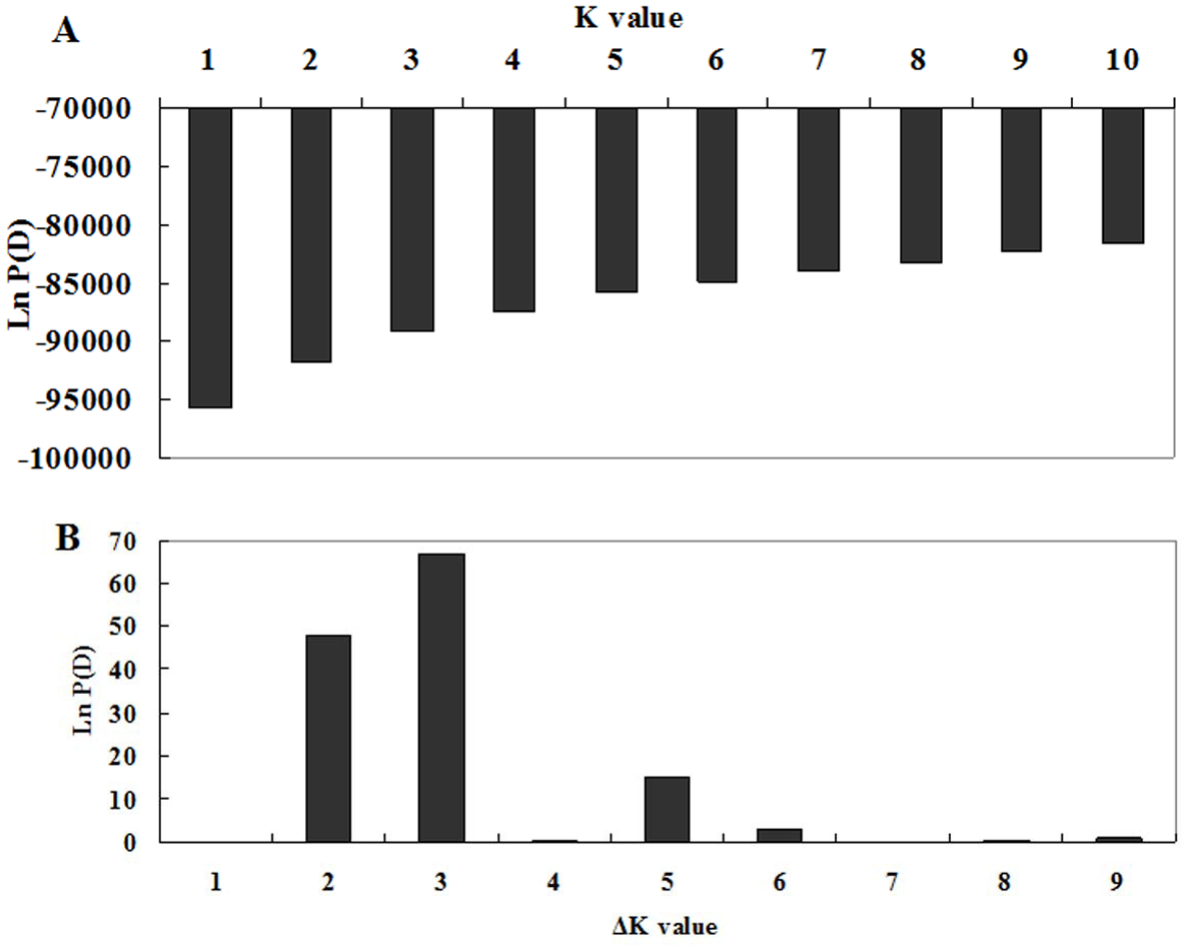
Calculation of true K of tall fescue accessions and (A) Evolution of the average logarithm probability of the data likelihoods (LnP(D)) for tall fescue genotypes; (B) Magnitude of Δk for each K value according to Evanno *et al*. [38].

According to STRUCTURE analysis results based on Bayesian clustering approach model, a significant population structure was detected among the 115 gene bank accessions. Three structure groups (GI, GII and GIII) were identified in the collection of 115 tall fescue accessions (Fig 2).

### Association analysis

Association mapping was conducted to find SSR markers potentially associated or linked with agronomic traits. Many marker alleles were associated with agronomic traits in our study. Table 2 gave an overview of single-associated markers with their genome positions and association with agronomic traits. Our association mapping has identified 41 single-associated SSR markers associated with agronomic traits (*P* < 0.05). With regard to agronomic traits, twelve single-associated markers were associated with PH (with the percentage of the total variation explained by each marker ranged from 3.7% to 7.3%), six single-associated markers were associated with BCS (with the percentage of the total variation explained by each marker ranged from 3.6% to 9.7%), eight single-associated markers were associated with SW (with the percentage of the total variation explained by each marker ranged from 3.4% to 11.6%), five single-associated markers were associated with SC (with the percentage of the total variation explained by each marker ranged from 3.7% to 7.9%), seven single-associated markers were associated with SCP (with the percentage of the total variation explained by each marker ranged from 3.3% to 7.4%), three single-associated markers were associated with SL (with the percentage of the total variation explained by each marker ranged from 5.6% to 6.3%). Especially, we observed that the genetic variation of SW was explained 11.6% by M37 marker.

**Table 2:** Association of SSR markers with percentage of reduction of single agronomic traits of tall fescue accessions by corrected *P* values (*P*<3.5×10^−4^).

Trait	Marker	Marker___F	Marker___P	MarkerR2	Trait	Marker	Marker___F	Marker___P	MarkerR2
**PH**	M181	8.83808	0.003612	0.072857	SW	M37	7.856043	0.000644	0.115551
**PH**	M214	4.048976	0.020079	0.067731	SW	M227	4.602934	0.01201	0.071367
**PH**	M48	3.893267	0.023219	0.065297	SW	M159	3.864508	0.023852	0.060663
**PH**	M112	4.977686	0.027668	0.042388	SW	M144	5.063413	0.026389	0.040307
**PH**	M238	3.431016	0.035824	0.057995	SW	M202	3.421642	0.036142	0.054115
**PH**	M213	3.428832	0.035898	0.057961	SW	M20	4.455876	0.037006	0.035656
**PH**	M116	4.366932	0.038907	0.037382	SW	M158	3.327028	0.039513	0.052703
**PH**	M140	4.366932	0.038907	0.037382	SW	M110	4.285284	0.040743	0.034341
**PH**	M215	3.292512	0.040821	0.055785	SCP	M121	4.835937	0.009689	0.0744
**PH**	M217	3.288661	0.04097	0.055724	SCP	M118	5.616214	0.019505	0.044325
**PH**	M216	3.288352	0.040982	0.055719	SCP	M221	4.054471	0.019976	0.063196
**PH**	M218	3.252663	0.042387	0.055148	SCP	M7	3.477188	0.0343	0.054728
**BCS**	M165	12.25476	0.000667	0.096897	SCP	M176	4.138552	0.044279	0.033078
**BCS**	M49	5.042963	0.008012	0.081835	SCP	M24	4.063729	0.046207	0.032501
**BCS**	M30	4.559593	0.012501	0.074587	SCP	M249	3.108965	0.048557	0.04924
**BCS**	M219	3.995293	0.021109	0.065976	SC	M32	4.778289	0.010217	0.078779
**BCS**	M170	4.36079	0.039042	0.036819	SC	M99	4.004935	0.02092	0.066887
**BCS**	M113	4.254887	0.04145	0.035958	SC	M179	4.705231	0.032185	0.040067
**SL**	M173	6.659323	0.011157	0.05594	SC	M100	3.447404	0.035275	0.05812
**SL**	M251	4.045833	0.020138	0.067725	SC	M104	4.315732	0.040048	0.036873
**SL**	M25	3.769239	0.026075	0.06339					

Table 3 gives an overview of multi-associated markers with their genome positions and association with agronomic traits. 20 multi-associations were determined between the SSR markers and the seven agronomic traits at the level of *P* < 0.05, and nine multi-associated markers were associated with more than one trait. For example, M3 and M4 were associated with three traits (BCS, SW, and SC for M3; BCS, SW and SC for M4). Four markers, M1, M2, M35 and M54, were associated with both BCS and SC. Meanwhile, three markers, M19, M40 and M193 were associated with two traits (PH and PD for M19; SW and SCP for M40; PH and SL for M193). With regard to agronomic traits, the number of loci associated with each trait ranged from one (PD SL and SCP) to six (BCS and SC). Two markers were associated with PH (with the percentage of the total variation explained by each marker with 4.3% and 5.3%), six markers were associated with BCS and SC (BCS variation explained by each marker ranged from 5.8% to 12.6%; SC variation explained by each marker ranged from 5.7% to 7.4%), three markers were associated with SW (explained by each marker ranged from 3.2% to 5.8%), and only one marker was associated with SL, PD, and SCP (SL was explained by M193 with 6.8%; PD was explained by M19 with 4.6%; SCP was explained by M40 with 2.1%). Especially, the BCS variation was explained 12.6% by M1 marker.

**Table 3:** Association of SSR markers with percentage of reduction of complex agronomic traits of tall fescue accessions by corrected *P* values (*P*<3.5×10^−4^).

Trait	Marker	Marker___F	Marker___P	MarkerR2	Trait	Marker	Marker___F	Marker___P	MarkerR2
**PH**	M193	3.099216	0.049008	0.052684	SCP	M40	5.494133	0.020843	0.043406
**PH**	M19	4.991568	0.027457	0.042501	SW	M4	3.681884	0.0283	0.057975
**BCS**	M1	8.165018	0.000492	0.126001	SW	M3	3.294229	0.040755	0.052213
**BCS**	M3	7.245904	0.001102	0.113456	SW	M40	3.922434	0.050098	0.031531
**BCS**	M2	6.991006	0.001382	0.109911	SC	M54	4.483199	0.013416	0.074277
**BCS**	M4	6.962296	0.001417	0.10951	SC	M4	3.586511	0.03095	0.060323
**BCS**	M54	5.054627	0.007927	0.082009	SC	M1	3.559831	0.031735	0.059901
**BCS**	M35	3.487192	0.033979	0.058081	SC	M3	3.494609	0.033742	0.058868
**SL**	M193	4.064402	0.019792	0.068015	SC	M2	3.486885	0.033989	0.058746
**PD**	M19	5.464602	0.021181	0.046455	SC	M35	3.365868	0.038092	0.056824

## Discussion

### Genetic variation in agronomic traits of tall fescue accessions

The estimation of genetic variation with species is crucial for their conservation and utilization [42]. Genetic diversity is necessary to sustain the productivity of forage as it furnishes new genes for yield, adaption, disease resistance, high value uses and characters [43]. In order to maintain, evaluate and utilize germplasm effectively, it is important to investigate the extent of genotypic diversity available. Smith and Smith [44] reported agro-morphological characterization as an important first step in description and classification of crop germplasm because a breeding program mainly depends upon the magnitude of genetic variability [8]. There have been a lot of successful examples in this area, such as rice [45], maize [46], wheat [47] and pea [48]. These studies indicated that high diversity in morphological characters could be a useful tool for germplasm collection. Plant height, spike length, spikelet number, spike number per plant and spike weight are the major components of plant yield used as selection criteria in breeding [49]. Among the 115 tall fescue accessions, significant genetic variation occurred in the agronomic traits were revealed in our study. This was consistent with the observations in perennial ryegrass [50], and tall fescue [51].

CV is a helpful indicator for selection of phenotypic variants of interest for breeding purpose. SCP and SW exhibited the greater CV, 55.6% and 48.3%, respectively, which in turn suggests good potential for selection in a breeding program. The SCP and SW are two determinative factors for high yield [52]. Thus, the observed germplasm as parent material showing the most interesting characteristics for a future breeding programe could be selected, as the yield potential can be enhanced through regulating SCP and SW. On the contrary, the lowest levels of variability found in BCP among all the detected traits indicate their low efficiency to evaluate genetic variability in tall fescue. Additionally, no significant difference of CV between PH, SL, PD and SC was found. Overall, the difference cultivar variation may be due to the complex inheritance of agronomic traits or the different geographical origins [52].

### Population structure

The population structure separation from the genetic linkage as causes for marker-trait association is considered to be the key factor in association analysis. Accessions with diverse geographical origins, the germplasm panel may contain either population structure (associated with local adaptation of diversifying selection), or familiar relatedness (from the recent co-ancestry) [26]. As population structure is universal among organisms [53], the population study might easily detect the false-positive results if without correctly controlled in associated mapping [25]. Population structure could explain a large fraction of phenotypic variance among the strains, the power to identify statistically significant quantitative loci decreases [22]. The presence of subpopulations can result in spurious associations due to confounding of unlinked markers with phenotypic variation [54]. Flint-Garcia et al. [25] reported that the observed traits were highly correlated with population structure, and 33% to 35% of variation of phenotypic traits about flowing time in a diverse maize panel would be attributed to population structure. Therefore, if the subpopulation structure was ignored, spurious associations may be identified at other loci that were differentially distributed among subpopulations. To eliminate the negative effects by population structure in association analysis, 115 accessions from different origins were selected to maintain diverse geographical origins representation. Taken the geographical origins and the history utilization of tall fescue into account, familiar relatedness, or/and population structure must be existed in the observed tall fescue accessions in our study.

With regard to our research, population structure was observed among 115 tall fescue accessions. Three groups were divided among the entire tall fescue population. The main subpopulation was consisted of 87 wild accessions from European, America, and all commercial cultivars. The wild accessions from Africa and Asia were separated into the second subpopulation. The third subpopulation was only consisted 11 wild accessions, with 5 accessions from Africa. According to the previous studies, we can attribute this phenomenon to different evolutionary paths and ecological adaptation [55, 56].

### Association analysis

As the successful utilization in maize, durum wheat, spring wheat, sugar beet, rice, grape and forage grasses [57, 27], the significant marker-trait associations would provide information on the location of the genome regions controlling traits of interest. In turn, this information would assist in making the best use of these genetic resources. Notably, many association mapping studies conducted on plants in the last few years have used small population sizes (i. e., smaller than 100 individuals) [58–60]. However, the population sizes of 100 are sufficient to detect QTL using GWAS when mapping within breeding programs [61].

Association mapping presents the opportunities to observe the genetic variation in natural population [62]. Zhang et al. [63] identified association mapping between agronomic traits and SSR markers in rice (*Oryza sativa* L.), and reported that 76 significant (*P*<0.5) trait—marker associations were detected, and 11 significant associations had >10% explained ratio of genetic variation. 390 linseed (*Linum usitatissimum* L.) and 464 SSR markers were employed by Soto-Cerda et al. [64] for association mapping, and the results indicated that 12 significant marker—trait associations were identified. Association mapping was also applied to barley (*Hordeum vulgare* L.) germplasm, and seven marker locis were detected that associated with plant height, and only one marker was observed that associated with stem diameter. The percentage of the total variation explained by each marker ranged from 4.59% (*HVM2* associated with plant height) to 17.48% (*Bmac* 90 associated with density of main spike) [21]. Notably, little is known about the association of SSR loci with agronomic traits in tall fescue.

With regard to agronomic traits analyzed in the present study, the following nine markers were associated with more than two agronomic traits: M1 and M2 (was associated with BCS and SC); M3 and M4 (was associated with BCS, SC and SW); M19 (was associated with PH and PD); M35 (was associated with BCS and SC); M40 (was associated with SW and SCP); M54 (was associated with BCS and SC); M193 (was associated with PH and SL). These markers were considered for greatly utilization to correlate agronomic traits that have not been reported, alternatively, were confirmed the suspect of the correlation with agronomic traits, and these correlation need more investigation. Notably, M3 and M4 were associated with BCP, SW and SC, suggesting that these three traits might correlate to each other. All the six markers, M1, M2, M3, M4, M35 and M54 were associated with BCS and SC, suggesting that these two traits might have significant correlation. Four significant associations had > 10% explained ratio of genetic variation, and these markers should be well applied in future tall fescue breeding.

In summary, association mapping has become a powerful tool for identifying genes and markers linked to agronomic traits. Large variation in SCP, SW was found among accessions of tall fescue. Three subpopulations were identified in the collection but no obvious relative kinship (*K*) was found. The GLM model was the best model to describe association between SSR and agronomic traits. Fifty-one SSR markers associated with agronomic traits were determined. It is interesting to observe that nine markers (M1, M2, M3, M4, M19, M35, M40, M54, and M193) were associated with more than two agronomic traits. Notably, BCS was explained by four markers (M1, M2, M3, and M4) exceed 10%. It is necessary for tall fescue selection breeding because these markers would enhance efficiency in desirable allele selection.

## Supporting Information

S1 File Table A in S1 File. The code, name, origin and status of 115 tall fescue accessions used in this study. Table B in S1 File. Characteristics of the 90 SSR primers used for the genetic relationship analysis in 115 tall fescue accessions.
(DOC)
