# Supplementary material for: Association Analysis of Simple Sequence Repeat (SSR) Markers with Agronomic Traits in Tall Fescue (Festuca arundinacea Schreb.)
Source: PLoS One. 2015 Jul 17;10(7):e0133054. doi: 10.1371/journal.pone.0133054 (PMC4505963; doi:10.1371/journal.pone.0133054)
Supplement: S1 File — Table A in S1 File. The code, name, origin and status of 115 tall fescue accessions used in this study. Table B in S1 File. Characteristics of the 90 SSR primers used for the genetic relationship analysis in 115 tall fescue accessions. (DOC) [file pone.0133054.s001.doc]

Table A The code, name, origin and status of 115 tall fescue accessions used in this study

| Code | Name | Origin | Status | Code | Name | Origin | Status |
| --- | --- | --- | --- | --- | --- | --- | --- |
| C1 | AST-1 | USDA | Cultivar | C59 | PI 598491 | European | Wild |
| C2 | Firenza | USDA | Cultivar | C60 | PI 442490 | European | Wild |
| C3 | Matador | USDA | Cultivar | C61 | PI 235125 | European | Wild |
| C4 | Coyote II | USDA | Cultivar | C62 | PI 283304 | European | Wild |
| C5 | Pixie | USDA | Cultivar | C63 | PI 423045 | European | Wild |
| C6 | SH3 | USDA | Cultivar | C64 | PI 512315 | European | Wild |
| C7 | Rembrandt | USDA | Cultivar | C65 | PI 598930 | European | Wild |
| C8 | SR8650 | USDA | Cultivar | C66 | PI 598493 | European | Wild |
| C9 | Tomahawk GT | USDA | Cultivar | C67 | PI 231563 | European | Wild |
| C10 | Grand II | USDA | Cultivar | C68 | PI 249738 | European | Wild |
| C11 | Tahoe II | USDA | Cultivar | C69 | PI 311044 | European | Wild |
| C12 | Lindergh | USDA | Cultivar | C70 | PI 595072 | European | Wild |
| C13 | LS-11 | USDA | Cultivar | C71 | PI 610933 | European | Wild |
| C14 | 3rd-Millennium | USDA | Cultivar | C72 | PI 527504 | European | Wild |
| C15 | Smirna | USDA | Cultivar | C73 | PI 636601 | European | Wild |
| C16 | Justice | USDA | Cultivar | C74 | PI 234881 | European | Wild |
| C17 | Millennium | USDA | Cultivar | C75 | PI 257742 | European | Wild |
| C18 | Endeavor | USDA | Cultivar | C76 | PI 184041 | European | Wild |
| C19 | Stone wall | USDA | Cultivar | C77 | PI 577082 | European | Wild |
| C20 | Silverado II | USDA | Cultivar | C78 | PI 423090 | European | Wild |
| C21 | ATM | USDA | Cultivar | C79 | PI 504538 | European | Wild |
| C22 | Barlexas | USDA | Cultivar | C80 | PI 234883 | European | Wild |
| C23 | Wolfpack | USDA | Cultivar | C81 | PI 274617 | European | Wild |
| C24 | Davinci | USDA | Cultivar | C82 | PI 255874 | European | Wild |
| C25 | Focus | USDA | Cultivar | C83 | PI 598496 | European | Wild |
| C26 | PI 574522 | America | Wild | C84 | PI 422638 | European | Wild |
| C27 | PI 636597 | America | Wild | C85 | PI 577081 | European | Wild |
| C28 | PI 634238 | America | Wild | C86 | PI 235036 | European | Wild |
| C29 | PI 561430 | America | Wild | C87 | PI 283281 | European | Wild |
| C30 | PI 601447 | America | Wild | C88 | PI 283287 | European | Wild |
| C31 | PI 600739 | America | Wild | C89 | PI 636532 | Africa | Wild |
| C32 | PI 538330 | America | Wild | C90 | PI 598860 | Africa | Wild |
| C33 | PI 531230 | America | Wild | C91 | PI 610909 | Africa | Wild |
| C34 | PI 578717 | America | Wild | C92 | PI 610951 | Africa | Wild |
| C35 | PI 655112 | America | Wild | C93 | PI 208679 | Africa | Wild |
| C36 | PI 469244 | America | Wild | C94 | PI 208681 | Africa | Wild |
| C37 | PI 608024 | America | Wild | C95 | PI 224975 | Africa | Wild |
| C38 | PI 600801 | America | Wild | C96 | PI 655104 | Asia | Wild |
| C39 | PI 203728 | America | Wild | C97 | PI 388898 | Asia | Wild |
| C40 | PI 578718 | America | Wild | C98 | PI 499494 | Asia | Wild |
| C41 | PI 655113 | America | Wild | C99 | PI 502373 | Asia | Wild |
| C42 | PI 578714 | America | Wild | C100 | PI 619025 | Asia | Wild |
| C43 | PI 608025 | America | Wild | C101 | PI 499495 | Asia | Wild |
| C44 | PI 538006 | America | Wild | C102 | PI388897 | Asia | Wild |
| C45 | PI 508603 | America | Wild | C103 | PI 269894 | Asia | Wild |
| C46 | PI 583822 | America | Wild | C104 | PI 174210 | Asia | Wild |
| C47 | PI 578719 | America | Wild | C105 | PI 547396 | Asia | Wild |
| C48 | PI 601106 | America | Wild | C106 | PI 440345 | Asia | Wild |
| C49 | PI 632516 | America | Wild | C107 | PI 200339 | Asia | Wild |
| C50 | PI 608808 | America | Wild | C108 | PI 598574 | Asia | Wild |
| C51 | PI 596701 | America | Wild | C109 | PI 438521 | Asia | Wild |
| C52 | PI 578724 | America | Wild | C110 | PI 211032 | Asia | Wild |
| C53 | PI 559374 | America | Wild | C111 | PI 505833 | Asia | Wild |
| C54 | PI 601227 | America | Wild | C112 | PI 618971 | Asia | Wild |
| C55 | PI 608787 | America | Wild | C113 | PI 618973 | Asia | Wild |
| C56 | PI 427127 | America | Wild | C114 | PI 619005 | Asia | Wild |
| C57 | PI 512305 | European | Wild | C115 | PI 380844 | Asia | Wild |
| C58 | PI 577094 | European | Wild |  |  |  |  |

Table B Characteristics of the 90 SSR primers used for the genetic relationship analysis in 115 tall fescue accessions

| Marker  ID | Marker Name | Forward Primer | Reverse Primer | Size  Range | Number of Allele |
| --- | --- | --- | --- | --- | --- |
| M1 | NFA001 | ctgctgctgccaagaaag t | taaggg gag cgagctacaga | 187-206 | 4 |
| M2 | NFA001 | ctgctgctgccaagaaag t | taaggg gag cgagctacaga | 187-206 | 4 |
| M3 | NFA001 | ctgctgctgccaagaaag t | taaggg gag cgagctacaga | 187-206 | 4 |
| M4 | NFA001 | ctgctgctgccaagaaag t | taaggg gag cgagctacaga | 187-206 | 4 |
| M5 | NFA006 | AAGCGGGAGGAGAGATGG | CACCACGACGTCGCTCTC | 205-213 | 4 |
| M6 | NFA006 | AAGCGGGAGGAGAGATGG | CACCACGACGTCGCTCTC | 205-213 | 4 |
| M7 | NFA006 | AAGCGGGAGGAGAGATGG | CACCACGACGTCGCTCTC | 205-213 | 4 |
| M8 | NFA006 | AAGCGGGAGGAGAGATGG | CACCACGACGTCGCTCTC | 205-213 | 4 |
| M9 | NFA012 | TTGCCAGGAAATTTTGCTCT | ATGGTATTGGCAGCAAGAGG | 274-309 | 10 |
| M10 | NFA012 | TTGCCAGGAAATTTTGCTCT | ATGGTATTGGCAGCAAGAGG | 274-309 | 10 |
| M11 | NFA012 | TTGCCAGGAAATTTTGCTCT | ATGGTATTGGCAGCAAGAGG | 274-309 | 10 |
| M12 | NFA012 | TTGCCAGGAAATTTTGCTCT | ATGGTATTGGCAGCAAGAGG | 274-309 | 10 |
| M13 | NFA012 | TTGCCAGGAAATTTTGCTCT | ATGGTATTGGCAGCAAGAGG | 274-309 | 10 |
| M14 | NFA012 | TTGCCAGGAAATTTTGCTCT | ATGGTATTGGCAGCAAGAGG | 274-309 | 10 |
| M15 | NFA012 | TTGCCAGGAAATTTTGCTCT | ATGGTATTGGCAGCAAGAGG | 274-309 | 10 |
| M16 | NFA012 | TTGCCAGGAAATTTTGCTCT | ATGGTATTGGCAGCAAGAGG | 274-309 | 10 |
| M17 | NFA012 | TTGCCAGGAAATTTTGCTCT | ATGGTATTGGCAGCAAGAGG | 274-309 | 10 |
| M18 | NFA012 | TTGCCAGGAAATTTTGCTCT | ATGGTATTGGCAGCAAGAGG | 274-309 | 10 |
| M19 | NFA013 | TCATTGTGTTCGCTCTCCTG | CCTTCGTCGCCATGGTAG | 226-271 | 13 |
| M20 | NFA013 | TCATTGTGTTCGCTCTCCTG | CCTTCGTCGCCATGGTAG | 226-271 | 13 |
| M21 | NFA013 | TCATTGTGTTCGCTCTCCTG | CCTTCGTCGCCATGGTAG | 226-271 | 13 |
| M22 | NFA013 | TCATTGTGTTCGCTCTCCTG | CCTTCGTCGCCATGGTAG | 226-271 | 13 |
| M23 | NFA013 | TCATTGTGTTCGCTCTCCTG | CCTTCGTCGCCATGGTAG | 226-271 | 13 |
| M24 | NFA013 | TCATTGTGTTCGCTCTCCTG | CCTTCGTCGCCATGGTAG | 226-271 | 13 |
| M25 | NFA013 | TCATTGTGTTCGCTCTCCTG | CCTTCGTCGCCATGGTAG | 226-271 | 13 |
| M26 | NFA013 | TCATTGTGTTCGCTCTCCTG | CCTTCGTCGCCATGGTAG | 226-271 | 13 |
| M27 | NFA013 | TCATTGTGTTCGCTCTCCTG | CCTTCGTCGCCATGGTAG | 226-271 | 13 |
| M28 | NFA013 | TCATTGTGTTCGCTCTCCTG | CCTTCGTCGCCATGGTAG | 226-271 | 13 |
| M29 | NFA013 | TCATTGTGTTCGCTCTCCTG | CCTTCGTCGCCATGGTAG | 226-271 | 13 |
| M30 | NFA013 | TCATTGTGTTCGCTCTCCTG | CCTTCGTCGCCATGGTAG | 226-271 | 13 |
| M31 | NFA013 | TCATTGTGTTCGCTCTCCTG | CCTTCGTCGCCATGGTAG | 226-271 | 13 |
| M32 | NFA017 | GATGGACGAAGGCTTCTTTG | AGCCGAACCTGAACTCAGAC | 176-198 | 8 |
| M33 | NFA017 | GATGGACGAAGGCTTCTTTG | AGCCGAACCTGAACTCAGAC | 176-198 | 8 |
| M34 | NFA017 | GATGGACGAAGGCTTCTTTG | AGCCGAACCTGAACTCAGAC | 176-198 | 8 |
| M35 | NFA017 | GATGGACGAAGGCTTCTTTG | AGCCGAACCTGAACTCAGAC | 176-198 | 8 |
| M36 | NFA017 | GATGGACGAAGGCTTCTTTG | AGCCGAACCTGAACTCAGAC | 176-198 | 8 |
| M37 | NFA017 | GATGGACGAAGGCTTCTTTG | AGCCGAACCTGAACTCAGAC | 176-198 | 8 |
| M38 | NFA017 | GATGGACGAAGGCTTCTTTG | AGCCGAACCTGAACTCAGAC | 176-198 | 8 |
| M39 | NFA017 | GATGGACGAAGGCTTCTTTG | AGCCGAACCTGAACTCAGAC | 176-198 | 8 |
| M40 | NFA019 | TGGATTTGCAATTAGCCTCA | GCTCGTGTATGGCCTTCAAT | 168-182 | 8 |
| M41 | NFA019 | TGGATTTGCAATTAGCCTCA | GCTCGTGTATGGCCTTCAAT | 168-182 | 8 |
| M42 | NFA019 | TGGATTTGCAATTAGCCTCA | GCTCGTGTATGGCCTTCAAT | 168-182 | 8 |
| M43 | NFA019 | TGGATTTGCAATTAGCCTCA | GCTCGTGTATGGCCTTCAAT | 168-182 | 8 |
| M44 | NFA019 | TGGATTTGCAATTAGCCTCA | GCTCGTGTATGGCCTTCAAT | 168-182 | 8 |
| M45 | NFA019 | TGGATTTGCAATTAGCCTCA | GCTCGTGTATGGCCTTCAAT | 168-182 | 8 |
| M46 | NFA019 | TGGATTTGCAATTAGCCTCA | GCTCGTGTATGGCCTTCAAT | 168-182 | 8 |
| M47 | NFA019 | TGGATTTGCAATTAGCCTCA | GCTCGTGTATGGCCTTCAAT | 168-182 | 8 |
| M48 | NFA020 | GCACGAGGCTCTTTCCTCTA | GGTGCTTGGCCTTCTTCC | 289-314 | 10 |
| M49 | NFA020 | GCACGAGGCTCTTTCCTCTA | GGTGCTTGGCCTTCTTCC | 289-314 | 10 |
| M50 | NFA020 | GCACGAGGCTCTTTCCTCTA | GGTGCTTGGCCTTCTTCC | 289-314 | 10 |
| M51 | NFA020 | GCACGAGGCTCTTTCCTCTA | GGTGCTTGGCCTTCTTCC | 289-314 | 10 |
| M52 | NFA020 | GCACGAGGCTCTTTCCTCTA | GGTGCTTGGCCTTCTTCC | 289-314 | 10 |
| M53 | NFA020 | GCACGAGGCTCTTTCCTCTA | GGTGCTTGGCCTTCTTCC | 289-314 | 10 |
| M54 | NFA020 | GCACGAGGCTCTTTCCTCTA | GGTGCTTGGCCTTCTTCC | 289-314 | 10 |
| M55 | NFA020 | GCACGAGGCTCTTTCCTCTA | GGTGCTTGGCCTTCTTCC | 289-314 | 10 |
| M56 | NFA020 | GCACGAGGCTCTTTCCTCTA | GGTGCTTGGCCTTCTTCC | 289-314 | 10 |
| M57 | NFA020 | GCACGAGGCTCTTTCCTCTA | GGTGCTTGGCCTTCTTCC | 289-314 | 10 |
| M58 | NFA022 | ATGATGTCCGAGGAGGAGAA | CATCATGATCCAGTGCCTTG | 170-224 | 11 |
| M59 | NFA022 | ATGATGTCCGAGGAGGAGAA | CATCATGATCCAGTGCCTTG | 170-224 | 11 |
| M60 | NFA022 | ATGATGTCCGAGGAGGAGAA | CATCATGATCCAGTGCCTTG | 170-224 | 11 |
| M61 | NFA022 | ATGATGTCCGAGGAGGAGAA | CATCATGATCCAGTGCCTTG | 170-224 | 11 |
| M62 | NFA022 | ATGATGTCCGAGGAGGAGAA | CATCATGATCCAGTGCCTTG | 170-224 | 11 |
| M63 | NFA022 | ATGATGTCCGAGGAGGAGAA | CATCATGATCCAGTGCCTTG | 170-224 | 11 |
| M64 | NFA022 | ATGATGTCCGAGGAGGAGAA | CATCATGATCCAGTGCCTTG | 170-224 | 11 |
| M65 | NFA022 | ATGATGTCCGAGGAGGAGAA | CATCATGATCCAGTGCCTTG | 170-224 | 11 |
| M66 | NFA022 | ATGATGTCCGAGGAGGAGAA | CATCATGATCCAGTGCCTTG | 170-224 | 11 |
| M67 | NFA022 | ATGATGTCCGAGGAGGAGAA | CATCATGATCCAGTGCCTTG | 170-224 | 11 |
| M68 | NFA022 | ATGATGTCCGAGGAGGAGAA | CATCATGATCCAGTGCCTTG | 170-224 | 11 |
| M69 | NFA023 | AGTCGGTGGTGAAGCTGAAG | TACAACTAGGGGGCTGGTCA | 174-209 | 8 |
| M70 | NFA023 | AGTCGGTGGTGAAGCTGAAG | TACAACTAGGGGGCTGGTCA | 174-209 | 8 |
| M71 | NFA023 | AGTCGGTGGTGAAGCTGAAG | TACAACTAGGGGGCTGGTCA | 174-209 | 8 |
| M72 | NFA023 | AGTCGGTGGTGAAGCTGAAG | TACAACTAGGGGGCTGGTCA | 174-209 | 8 |
| M73 | NFA023 | AGTCGGTGGTGAAGCTGAAG | TACAACTAGGGGGCTGGTCA | 174-209 | 8 |
| M74 | NFA023 | AGTCGGTGGTGAAGCTGAAG | TACAACTAGGGGGCTGGTCA | 174-209 | 8 |
| M75 | NFA023 | AGTCGGTGGTGAAGCTGAAG | TACAACTAGGGGGCTGGTCA | 174-209 | 8 |
| M76 | NFA023 | AGTCGGTGGTGAAGCTGAAG | TACAACTAGGGGGCTGGTCA | 174-209 | 8 |
| M77 | NFA027 | CGAGGTCTCAATCCTCCATT | GACAGAGACGACGACGACAT | 147-194 | 14 |
| M78 | NFA027 | CGAGGTCTCAATCCTCCATT | GACAGAGACGACGACGACAT | 147-194 | 14 |
| M79 | NFA027 | CGAGGTCTCAATCCTCCATT | GACAGAGACGACGACGACAT | 147-194 | 14 |
| M80 | NFA027 | CGAGGTCTCAATCCTCCATT | GACAGAGACGACGACGACAT | 147-194 | 14 |
| M81 | NFA027 | CGAGGTCTCAATCCTCCATT | GACAGAGACGACGACGACAT | 147-194 | 14 |
| M82 | NFA027 | CGAGGTCTCAATCCTCCATT | GACAGAGACGACGACGACAT | 147-194 | 14 |
| M83 | NFA027 | CGAGGTCTCAATCCTCCATT | GACAGAGACGACGACGACAT | 147-194 | 14 |
| M84 | NFA027 | CGAGGTCTCAATCCTCCATT | GACAGAGACGACGACGACAT | 147-194 | 14 |
| M85 | NFA027 | CGAGGTCTCAATCCTCCATT | GACAGAGACGACGACGACAT | 147-194 | 14 |
| M86 | NFA027 | CGAGGTCTCAATCCTCCATT | GACAGAGACGACGACGACAT | 147-194 | 14 |
| M87 | NFA027 | CGAGGTCTCAATCCTCCATT | GACAGAGACGACGACGACAT | 147-194 | 14 |
| M88 | NFA027 | CGAGGTCTCAATCCTCCATT | GACAGAGACGACGACGACAT | 147-194 | 14 |
| M89 | NFA027 | CGAGGTCTCAATCCTCCATT | GACAGAGACGACGACGACAT | 147-194 | 14 |
| M90 | NFA027 | CGAGGTCTCAATCCTCCATT | GACAGAGACGACGACGACAT | 147-194 | 14 |
| M91 | NFA029 | GGACGACATGTCTGTGCAGT | GCCTTGTCGCTGGCTACTC | 202-214 | 5 |
| M92 | NFA029 | GGACGACATGTCTGTGCAGT | GCCTTGTCGCTGGCTACTC | 202-214 | 5 |
| M93 | NFA029 | GGACGACATGTCTGTGCAGT | GCCTTGTCGCTGGCTACTC | 202-214 | 5 |
| M94 | NFA029 | GGACGACATGTCTGTGCAGT | GCCTTGTCGCTGGCTACTC | 202-214 | 5 |
| M95 | NFA029 | GGACGACATGTCTGTGCAGT | GCCTTGTCGCTGGCTACTC | 202-214 | 5 |
| M96 | NFA030 | AGTCGGTGGTGAAGCTGAAG | ACAACTAGGGGGCTGGTCA | 186-207 | 6 |
| M97 | NFA030 | AGTCGGTGGTGAAGCTGAAG | ACAACTAGGGGGCTGGTCA | 186-207 | 6 |
| M98 | NFA030 | AGTCGGTGGTGAAGCTGAAG | ACAACTAGGGGGCTGGTCA | 186-207 | 6 |
| M99 | NFA030 | AGTCGGTGGTGAAGCTGAAG | ACAACTAGGGGGCTGGTCA | 186-207 | 6 |
| M100 | NFA030 | AGTCGGTGGTGAAGCTGAAG | ACAACTAGGGGGCTGGTCA | 186-207 | 6 |
| M101 | NFA030 | AGTCGGTGGTGAAGCTGAAG | ACAACTAGGGGGCTGGTCA | 186-207 | 6 |
| M102 | NFA030 | AGTCGGTGGTGAAGCTGAAG | ACAACTAGGGGGCTGGTCA | 186-207 | 6 |
| M103 | NFA030 | AGTCGGTGGTGAAGCTGAAG | ACAACTAGGGGGCTGGTCA | 186-207 | 6 |
| M104 | NFA033 | CACGAGGGAACCAGACACAC | TCCCCTCTCCTCCTTCTCAT | 184-204 | 6 |
| M105 | NFA033 | CACGAGGGAACCAGACACAC | TCCCCTCTCCTCCTTCTCAT | 184-204 | 6 |
| M106 | NFA033 | CACGAGGGAACCAGACACAC | TCCCCTCTCCTCCTTCTCAT | 184-204 | 6 |
| M107 | NFA033 | CACGAGGGAACCAGACACAC | TCCCCTCTCCTCCTTCTCAT | 184-204 | 6 |
| M108 | NFA033 | CACGAGGGAACCAGACACAC | TCCCCTCTCCTCCTTCTCAT | 184-204 | 6 |
| M109 | NFA033 | CACGAGGGAACCAGACACAC | TCCCCTCTCCTCCTTCTCAT | 184-204 | 6 |
| M110 | NFA034 | GCTGGGTGTAGGGCTGTAAA | CTCCTTTCCATCACCTCTGG | 197-227 | 8 |
| M111 | NFA034 | GCTGGGTGTAGGGCTGTAAA | CTCCTTTCCATCACCTCTGG | 197-227 | 8 |
| M112 | NFA034 | GCTGGGTGTAGGGCTGTAAA | CTCCTTTCCATCACCTCTGG | 197-227 | 8 |
| M113 | NFA034 | GCTGGGTGTAGGGCTGTAAA | CTCCTTTCCATCACCTCTGG | 197-227 | 8 |
| M114 | NFA034 | GCTGGGTGTAGGGCTGTAAA | CTCCTTTCCATCACCTCTGG | 197-227 | 8 |
| M115 | NFA034 | GCTGGGTGTAGGGCTGTAAA | CTCCTTTCCATCACCTCTGG | 197-227 | 8 |
| M116 | NFA034 | GCTGGGTGTAGGGCTGTAAA | CTCCTTTCCATCACCTCTGG | 197-227 | 8 |
| M117 | NFA034 | GCTGGGTGTAGGGCTGTAAA | CTCCTTTCCATCACCTCTGG | 197-227 | 8 |
| M118 | NFA035 | TGCTAGCAGGGGTCTAAGGA | CACACGTACCACGTCTCCAT | 167-192 | 10 |
| M119 | NFA035 | TGCTAGCAGGGGTCTAAGGA | CACACGTACCACGTCTCCAT | 167-192 | 10 |
| M120 | NFA035 | TGCTAGCAGGGGTCTAAGGA | CACACGTACCACGTCTCCAT | 167-192 | 10 |
| M121 | NFA035 | TGCTAGCAGGGGTCTAAGGA | CACACGTACCACGTCTCCAT | 167-192 | 10 |
| M122 | NFA035 | TGCTAGCAGGGGTCTAAGGA | CACACGTACCACGTCTCCAT | 167-192 | 10 |
| M123 | NFA035 | TGCTAGCAGGGGTCTAAGGA | CACACGTACCACGTCTCCAT | 167-192 | 10 |
| M124 | NFA035 | TGCTAGCAGGGGTCTAAGGA | CACACGTACCACGTCTCCAT | 167-192 | 10 |
| M125 | NFA035 | TGCTAGCAGGGGTCTAAGGA | CACACGTACCACGTCTCCAT | 167-192 | 10 |
| M126 | NFA035 | TGCTAGCAGGGGTCTAAGGA | CACACGTACCACGTCTCCAT | 167-192 | 10 |
| M127 | NFA035 | TGCTAGCAGGGGTCTAAGGA | CACACGTACCACGTCTCCAT | 167-192 | 10 |
| M128 | NFA036 | AGAGGAAGAGCGAAAGAGCA | CCCTGGTACTCGTGGATGTT | 176-198 | 7 |
| M129 | NFA036 | AGAGGAAGAGCGAAAGAGCA | CCCTGGTACTCGTGGATGTT | 176-198 | 7 |
| M130 | NFA036 | AGAGGAAGAGCGAAAGAGCA | CCCTGGTACTCGTGGATGTT | 176-198 | 7 |
| M131 | NFA036 | AGAGGAAGAGCGAAAGAGCA | CCCTGGTACTCGTGGATGTT | 176-198 | 7 |
| M132 | NFA036 | AGAGGAAGAGCGAAAGAGCA | CCCTGGTACTCGTGGATGTT | 176-198 | 7 |
| M133 | NFA036 | AGAGGAAGAGCGAAAGAGCA | CCCTGGTACTCGTGGATGTT | 176-198 | 7 |
| M134 | NFA036 | AGAGGAAGAGCGAAAGAGCA | CCCTGGTACTCGTGGATGTT | 176-198 | 7 |
| M135 | NFA040 | TCATTGTGTTCGCTCTCCTG | CCTTCTTTGTCGCCATGGTA | 230-277 | 10 |
| M136 | NFA040 | TCATTGTGTTCGCTCTCCTG | CCTTCTTTGTCGCCATGGTA | 230-277 | 10 |
| M137 | NFA040 | TCATTGTGTTCGCTCTCCTG | CCTTCTTTGTCGCCATGGTA | 230-277 | 10 |
| M138 | NFA040 | TCATTGTGTTCGCTCTCCTG | CCTTCTTTGTCGCCATGGTA | 230-277 | 10 |
| M139 | NFA040 | TCATTGTGTTCGCTCTCCTG | CCTTCTTTGTCGCCATGGTA | 230-277 | 10 |
| M140 | NFA040 | TCATTGTGTTCGCTCTCCTG | CCTTCTTTGTCGCCATGGTA | 230-277 | 10 |
| M141 | NFA040 | TCATTGTGTTCGCTCTCCTG | CCTTCTTTGTCGCCATGGTA | 230-277 | 10 |
| M142 | NFA040 | TCATTGTGTTCGCTCTCCTG | CCTTCTTTGTCGCCATGGTA | 230-277 | 10 |
| M143 | NFA040 | TCATTGTGTTCGCTCTCCTG | CCTTCTTTGTCGCCATGGTA | 230-277 | 10 |
| M144 | NFA040 | TCATTGTGTTCGCTCTCCTG | CCTTCTTTGTCGCCATGGTA | 230-277 | 10 |
| M145 | NFA070 | TCCAGTTCAACTTCCAGCAA | CCCGTATTTTATCCTCGATCTG | 189-244 | 20 |
| M146 | NFA070 | TCCAGTTCAACTTCCAGCAA | CCCGTATTTTATCCTCGATCTG | 189-244 | 20 |
| M147 | NFA070 | TCCAGTTCAACTTCCAGCAA | CCCGTATTTTATCCTCGATCTG | 189-244 | 20 |
| M148 | NFA070 | TCCAGTTCAACTTCCAGCAA | CCCGTATTTTATCCTCGATCTG | 189-244 | 20 |
| M149 | NFA070 | TCCAGTTCAACTTCCAGCAA | CCCGTATTTTATCCTCGATCTG | 189-244 | 20 |
| M150 | NFA070 | TCCAGTTCAACTTCCAGCAA | CCCGTATTTTATCCTCGATCTG | 189-244 | 20 |
| M151 | NFA070 | TCCAGTTCAACTTCCAGCAA | CCCGTATTTTATCCTCGATCTG | 189-244 | 20 |
| M152 | NFA070 | TCCAGTTCAACTTCCAGCAA | CCCGTATTTTATCCTCGATCTG | 189-244 | 20 |
| M153 | NFA070 | TCCAGTTCAACTTCCAGCAA | CCCGTATTTTATCCTCGATCTG | 189-244 | 20 |
| M154 | NFA070 | TCCAGTTCAACTTCCAGCAA | CCCGTATTTTATCCTCGATCTG | 189-244 | 20 |
| M155 | NFA070 | TCCAGTTCAACTTCCAGCAA | CCCGTATTTTATCCTCGATCTG | 189-244 | 20 |
| M156 | NFA070 | TCCAGTTCAACTTCCAGCAA | CCCGTATTTTATCCTCGATCTG | 189-244 | 20 |
| M157 | NFA070 | TCCAGTTCAACTTCCAGCAA | CCCGTATTTTATCCTCGATCTG | 189-244 | 20 |
| M158 | NFA070 | TCCAGTTCAACTTCCAGCAA | CCCGTATTTTATCCTCGATCTG | 189-244 | 20 |
| M159 | NFA070 | TCCAGTTCAACTTCCAGCAA | CCCGTATTTTATCCTCGATCTG | 189-244 | 20 |
| M160 | NFA070 | TCCAGTTCAACTTCCAGCAA | CCCGTATTTTATCCTCGATCTG | 189-244 | 20 |
| M161 | NFA070 | TCCAGTTCAACTTCCAGCAA | CCCGTATTTTATCCTCGATCTG | 189-244 | 20 |
| M162 | NFA070 | TCCAGTTCAACTTCCAGCAA | CCCGTATTTTATCCTCGATCTG | 189-244 | 20 |
| M163 | NFA070 | TCCAGTTCAACTTCCAGCAA | CCCGTATTTTATCCTCGATCTG | 189-244 | 20 |
| M164 | NFA070 | TCCAGTTCAACTTCCAGCAA | CCCGTATTTTATCCTCGATCTG | 189-244 | 20 |
| M165 | NFA073 | TTCCTTCCTCTTTCCCGAAC | ATGGTCTCCCTCTGCTCGTA | 239-250 | 6 |
| M166 | NFA073 | TTCCTTCCTCTTTCCCGAAC | ATGGTCTCCCTCTGCTCGTA | 239-250 | 6 |
| M167 | NFA073 | TTCCTTCCTCTTTCCCGAAC | ATGGTCTCCCTCTGCTCGTA | 239-250 | 6 |
| M168 | NFA073 | TTCCTTCCTCTTTCCCGAAC | ATGGTCTCCCTCTGCTCGTA | 239-250 | 6 |
| M169 | NFA073 | TTCCTTCCTCTTTCCCGAAC | ATGGTCTCCCTCTGCTCGTA | 239-250 | 6 |
| M170 | NFA073 | TTCCTTCCTCTTTCCCGAAC | ATGGTCTCCCTCTGCTCGTA | 239-250 | 6 |
| M171 | NFA087 | GATCTTGCTCCACTCGATCTG | GCTCCAGTCATCTGAAATCTCC | 413-451 | 16 |
| M172 | NFA087 | GATCTTGCTCCACTCGATCTG | GCTCCAGTCATCTGAAATCTCC | 413-451 | 16 |
| M173 | NFA087 | GATCTTGCTCCACTCGATCTG | GCTCCAGTCATCTGAAATCTCC | 413-451 | 16 |
| M174 | NFA087 | GATCTTGCTCCACTCGATCTG | GCTCCAGTCATCTGAAATCTCC | 413-451 | 16 |
| M175 | NFA087 | GATCTTGCTCCACTCGATCTG | GCTCCAGTCATCTGAAATCTCC | 413-451 | 16 |
| M176 | NFA087 | GATCTTGCTCCACTCGATCTG | GCTCCAGTCATCTGAAATCTCC | 413-451 | 16 |
| M177 | NFA087 | GATCTTGCTCCACTCGATCTG | GCTCCAGTCATCTGAAATCTCC | 413-451 | 16 |
| M178 | NFA087 | GATCTTGCTCCACTCGATCTG | GCTCCAGTCATCTGAAATCTCC | 413-451 | 16 |
| M179 | NFA087 | GATCTTGCTCCACTCGATCTG | GCTCCAGTCATCTGAAATCTCC | 413-451 | 16 |
| M180 | NFA087 | GATCTTGCTCCACTCGATCTG | GCTCCAGTCATCTGAAATCTCC | 413-451 | 16 |
| M181 | NFA087 | GATCTTGCTCCACTCGATCTG | GCTCCAGTCATCTGAAATCTCC | 413-451 | 16 |
| M182 | NFA087 | GATCTTGCTCCACTCGATCTG | GCTCCAGTCATCTGAAATCTCC | 413-451 | 16 |
| M183 | NFA087 | GATCTTGCTCCACTCGATCTG | GCTCCAGTCATCTGAAATCTCC | 413-451 | 16 |
| M184 | NFA087 | GATCTTGCTCCACTCGATCTG | GCTCCAGTCATCTGAAATCTCC | 413-451 | 16 |
| M185 | NFA087 | GATCTTGCTCCACTCGATCTG | GCTCCAGTCATCTGAAATCTCC | 413-451 | 16 |
| M186 | NFA087 | GATCTTGCTCCACTCGATCTG | GCTCCAGTCATCTGAAATCTCC | 413-451 | 16 |
| M187 | NFA088 | ATTTAGAATGGAGACGCAGGAG | TAGTGTCTCTGTTGGGGATGTG | 214-265 | 19 |
| M188 | NFA088 | ATTTAGAATGGAGACGCAGGAG | TAGTGTCTCTGTTGGGGATGTG | 214-265 | 19 |
| M189 | NFA088 | ATTTAGAATGGAGACGCAGGAG | TAGTGTCTCTGTTGGGGATGTG | 214-265 | 19 |
| M190 | NFA088 | ATTTAGAATGGAGACGCAGGAG | TAGTGTCTCTGTTGGGGATGTG | 214-265 | 19 |
| M191 | NFA088 | ATTTAGAATGGAGACGCAGGAG | TAGTGTCTCTGTTGGGGATGTG | 214-265 | 19 |
| M192 | NFA088 | ATTTAGAATGGAGACGCAGGAG | TAGTGTCTCTGTTGGGGATGTG | 214-265 | 19 |
| M193 | NFA088 | ATTTAGAATGGAGACGCAGGAG | TAGTGTCTCTGTTGGGGATGTG | 214-265 | 19 |
| M194 | NFA088 | ATTTAGAATGGAGACGCAGGAG | TAGTGTCTCTGTTGGGGATGTG | 214-265 | 19 |
| M195 | NFA088 | ATTTAGAATGGAGACGCAGGAG | TAGTGTCTCTGTTGGGGATGTG | 214-265 | 19 |
| M196 | NFA088 | ATTTAGAATGGAGACGCAGGAG | TAGTGTCTCTGTTGGGGATGTG | 214-265 | 19 |
| M197 | NFA088 | ATTTAGAATGGAGACGCAGGAG | TAGTGTCTCTGTTGGGGATGTG | 214-265 | 19 |
| M198 | NFA088 | ATTTAGAATGGAGACGCAGGAG | TAGTGTCTCTGTTGGGGATGTG | 214-265 | 19 |
| M199 | NFA088 | ATTTAGAATGGAGACGCAGGAG | TAGTGTCTCTGTTGGGGATGTG | 214-265 | 19 |
| M200 | NFA088 | ATTTAGAATGGAGACGCAGGAG | TAGTGTCTCTGTTGGGGATGTG | 214-265 | 19 |
| M201 | NFA088 | ATTTAGAATGGAGACGCAGGAG | TAGTGTCTCTGTTGGGGATGTG | 214-265 | 19 |
| M202 | NFA088 | ATTTAGAATGGAGACGCAGGAG | TAGTGTCTCTGTTGGGGATGTG | 214-265 | 19 |
| M203 | NFA088 | ATTTAGAATGGAGACGCAGGAG | TAGTGTCTCTGTTGGGGATGTG | 214-265 | 19 |
| M204 | NFA088 | ATTTAGAATGGAGACGCAGGAG | TAGTGTCTCTGTTGGGGATGTG | 214-265 | 19 |
| M205 | NFA088 | ATTTAGAATGGAGACGCAGGAG | TAGTGTCTCTGTTGGGGATGTG | 214-265 | 19 |
| M206 | NFA090 | CCGTTTTGTTCTTCCCTTCTC | GCGAGAGAGTTTGGTGAGGATA | 326-353 | 10 |
| M207 | NFA090 | CCGTTTTGTTCTTCCCTTCTC | GCGAGAGAGTTTGGTGAGGATA | 326-353 | 10 |
| M208 | NFA090 | CCGTTTTGTTCTTCCCTTCTC | GCGAGAGAGTTTGGTGAGGATA | 326-353 | 10 |
| M209 | NFA090 | CCGTTTTGTTCTTCCCTTCTC | GCGAGAGAGTTTGGTGAGGATA | 326-353 | 10 |
| M210 | NFA090 | CCGTTTTGTTCTTCCCTTCTC | GCGAGAGAGTTTGGTGAGGATA | 326-353 | 10 |
| M211 | NFA090 | CCGTTTTGTTCTTCCCTTCTC | GCGAGAGAGTTTGGTGAGGATA | 326-353 | 10 |
| M212 | NFA090 | CCGTTTTGTTCTTCCCTTCTC | GCGAGAGAGTTTGGTGAGGATA | 326-353 | 10 |
| M213 | NFA090 | CCGTTTTGTTCTTCCCTTCTC | GCGAGAGAGTTTGGTGAGGATA | 326-353 | 10 |
| M214 | NFA090 | CCGTTTTGTTCTTCCCTTCTC | GCGAGAGAGTTTGGTGAGGATA | 326-353 | 10 |
| M215 | NFA090 | CCGTTTTGTTCTTCCCTTCTC | GCGAGAGAGTTTGGTGAGGATA | 326-353 | 10 |
| M216 | NFA112 | ATCATTTCGGTTTCAGGTCATC | AGGTAGCGACGAGGAGGAAC | 220-247 | 12 |
| M217 | NFA112 | ATCATTTCGGTTTCAGGTCATC | AGGTAGCGACGAGGAGGAAC | 220-247 | 12 |
| M218 | NFA112 | ATCATTTCGGTTTCAGGTCATC | AGGTAGCGACGAGGAGGAAC | 220-247 | 12 |
| M219 | NFA112 | ATCATTTCGGTTTCAGGTCATC | AGGTAGCGACGAGGAGGAAC | 220-247 | 12 |
| M220 | NFA112 | ATCATTTCGGTTTCAGGTCATC | AGGTAGCGACGAGGAGGAAC | 220-247 | 12 |
| M221 | NFA112 | ATCATTTCGGTTTCAGGTCATC | AGGTAGCGACGAGGAGGAAC | 220-247 | 12 |
| M222 | NFA112 | ATCATTTCGGTTTCAGGTCATC | AGGTAGCGACGAGGAGGAAC | 220-247 | 12 |
| M223 | NFA112 | ATCATTTCGGTTTCAGGTCATC | AGGTAGCGACGAGGAGGAAC | 220-247 | 12 |
| M224 | NFA112 | ATCATTTCGGTTTCAGGTCATC | AGGTAGCGACGAGGAGGAAC | 220-247 | 12 |
| M225 | NFA112 | ATCATTTCGGTTTCAGGTCATC | AGGTAGCGACGAGGAGGAAC | 220-247 | 12 |
| M226 | NFA112 | ATCATTTCGGTTTCAGGTCATC | AGGTAGCGACGAGGAGGAAC | 220-247 | 12 |
| M227 | NFA112 | ATCATTTCGGTTTCAGGTCATC | AGGTAGCGACGAGGAGGAAC | 220-247 | 12 |
| M228 | NFA113 | CTCGAAGTAGGCGGAGATGAC | AGTACGAGGAGGGGATGGAGAT | 190-283 | 12 |
| M229 | NFA113 | CTCGAAGTAGGCGGAGATGAC | AGTACGAGGAGGGGATGGAGAT | 190-283 | 12 |
| M230 | NFA113 | CTCGAAGTAGGCGGAGATGAC | AGTACGAGGAGGGGATGGAGAT | 190-283 | 12 |
| M231 | NFA113 | CTCGAAGTAGGCGGAGATGAC | AGTACGAGGAGGGGATGGAGAT | 190-283 | 12 |
| M232 | NFA113 | CTCGAAGTAGGCGGAGATGAC | AGTACGAGGAGGGGATGGAGAT | 190-283 | 12 |
| M233 | NFA113 | CTCGAAGTAGGCGGAGATGAC | AGTACGAGGAGGGGATGGAGAT | 190-283 | 12 |
| M234 | NFA113 | CTCGAAGTAGGCGGAGATGAC | AGTACGAGGAGGGGATGGAGAT | 190-283 | 12 |
| M235 | NFA113 | CTCGAAGTAGGCGGAGATGAC | AGTACGAGGAGGGGATGGAGAT | 190-283 | 12 |
| M236 | NFA113 | CTCGAAGTAGGCGGAGATGAC | AGTACGAGGAGGGGATGGAGAT | 190-283 | 12 |
| M237 | NFA113 | CTCGAAGTAGGCGGAGATGAC | AGTACGAGGAGGGGATGGAGAT | 190-283 | 12 |
| M238 | NFA113 | CTCGAAGTAGGCGGAGATGAC | AGTACGAGGAGGGGATGGAGAT | 190-283 | 12 |
| M239 | NFA113 | CTCGAAGTAGGCGGAGATGAC | AGTACGAGGAGGGGATGGAGAT | 190-283 | 12 |
| M240 | NFA129 | AACCTTGATGGGGCGTAAG | GCCGGAGTAGGAGGATTTTC | 216-251 | 11 |
| M241 | NFA129 | AACCTTGATGGGGCGTAAG | GCCGGAGTAGGAGGATTTTC | 216-251 | 11 |
| M242 | NFA129 | AACCTTGATGGGGCGTAAG | GCCGGAGTAGGAGGATTTTC | 216-251 | 11 |
| M243 | NFA129 | AACCTTGATGGGGCGTAAG | GCCGGAGTAGGAGGATTTTC | 216-251 | 11 |
| M244 | NFA129 | AACCTTGATGGGGCGTAAG | GCCGGAGTAGGAGGATTTTC | 216-251 | 11 |
| M245 | NFA129 | AACCTTGATGGGGCGTAAG | GCCGGAGTAGGAGGATTTTC | 216-251 | 11 |
| M246 | NFA129 | AACCTTGATGGGGCGTAAG | GCCGGAGTAGGAGGATTTTC | 216-251 | 11 |
| M247 | NFA129 | AACCTTGATGGGGCGTAAG | GCCGGAGTAGGAGGATTTTC | 216-251 | 11 |
| M248 | NFA129 | AACCTTGATGGGGCGTAAG | GCCGGAGTAGGAGGATTTTC | 216-251 | 11 |
| M249 | NFA129 | AACCTTGATGGGGCGTAAG | GCCGGAGTAGGAGGATTTTC | 216-251 | 11 |
| M250 | NFA129 | AACCTTGATGGGGCGTAAG | GCCGGAGTAGGAGGATTTTC | 216-251 | 11 |
| M251 | NFA135 | AGAGGGAGATACGTCAAGAGGA | CCGGTGAGTTGATAGTGGAGTC | 228-277 | 8 |
| M252 | NFA135 | AGAGGGAGATACGTCAAGAGGA | CCGGTGAGTTGATAGTGGAGTC | 228-277 | 8 |
| M253 | NFA135 | AGAGGGAGATACGTCAAGAGGA | CCGGTGAGTTGATAGTGGAGTC | 228-277 | 8 |
| M254 | NFA135 | AGAGGGAGATACGTCAAGAGGA | CCGGTGAGTTGATAGTGGAGTC | 228-277 | 8 |
| M255 | NFA135 | AGAGGGAGATACGTCAAGAGGA | CCGGTGAGTTGATAGTGGAGTC | 228-277 | 8 |
